# Multifaceted Social and Environmental Disruptions Impact on Smallholder Plantations' Resilience in Indonesia

**DOI:** 10.1155/2022/6360253

**Published:** 2022-12-13

**Authors:** Apri Andani, Irham Irham, Jamhari Jamhari, Any Suryantini

**Affiliations:** ^1^PhD Candidate in Agribusiness, Agricultural Doctorate Program, Faculty of Agriculture, Universitas Gadjah Mada, Yogyakarta, Indonesia; ^2^Agribusiness Department, Faculty of Agriculture, Universitas Bengkulu, Bengkulu, Indonesia; ^3^Scholarship Awardee of Indonesia Endowment Fund for Education (LPDP), Jakarta, Indonesia; ^4^Socioeconomics Department, Faculty of Agriculture, Universitas Gadjah Mada, Yogyakarta, Indonesia

## Abstract

As one of the most productive plantation producers in the world, Indonesia also faces rapid change in both social and environmental systems. These conditions are predicted to become more disruptive to the agricultural sector in the future. Therefore, understanding the impact of social and environmental disruption on smallholder plantations' resilience is vital to formulate a strategy for the sustainability of farmers' livelihoods in this country. Using survey data from 360 smallholding farmers in six villages from three districts in Bengkulu Province, Indonesia, the study deployed a multidimensional approach to assess smallholders' resilience to social and environmental disruption as well as towards economic dynamics. There are four dimensions of smallholder resilience, namely, the ability of adaptation, recoverability, anticipation, and farmers' innovation level. Social disruption was indicated by farmers' demography, epidemic/family health, social conflict, culture clash, and intention on land conversion. Meanwhile, environmental disruption was shown by natural catastrophe incidents, climate variations, environmentally unfriendly cultivation activities, and land fires. Since the resilience level was classified as binary, bivariate probit model was used in the analysis. The result shows that smallholder plantations in Bengkulu Indonesia are categorized as innovative, and recoverable, but less adaptive, and less anticipatory farmers. Overall, more than 50% of smallholder plantations are classified as less resilient smallholders. The statistical result empirically uncovers that the intentions of land conversion, climate change, and environmentally unfriendly farming activities statistically have a significant contribution to the reduction of smallholder plantations' resilience. Furthermore, the economic dynamisms such as lack of input availability, price volatility, demand uncertainty, and capital limitation have a significant negative impact on smallholder plantation resilience.

## 1. Introduction

Recently, both social and environmental disruption issues become popular among researchers. A rapid change in social aspects (including health, global pandemic, social conflict, and human behavior), and the rise of global environmental issues (including climate change, natural disasters, and eco-friendly industry), potentially tend to be disruptions for smallholder plantations. According to Sanchis & Poler, disruption can be an interrupting variable that results in deviations, inhibits, and forces businesses to make changes and adjustments [1]. A system is disrupted when the system must redesign its strategy to survive a change in the environment [2]. Merriam Webster furtherly explains that disruption is to cause (something) to be unable to maintain in the normal direction: to interrupt the normal progress or occupation of (something) [3]. In social subjects, disruption can be defined as a term used in sociology to describe the alteration, dysfunction, or breakdown of social life, often in a community setting [4]. This type of disruption implies a radical transformation [4]. Meanwhile, in environmental circumstances, disruption is referred to as ecological disturbance or ecological imbalance (including climate change, fires, flooding, insect and pest outbreaks, or earthquakes) that can cause environmental stress, and largely impact the ecosystem or natural resources [5]. This disruption potentially has a direct and significant impact on the agricultural system. Environmental disruptions can be caused by natural incidents or human activity [6]. Thus, in this research, we propose disruption as an event or any change, disturbance, interruption, or distraction, in a social and environmental term, which forces a system (smallholder plantations) to change its regular practices and then find a new strategy to survive.

**Figure 1:**
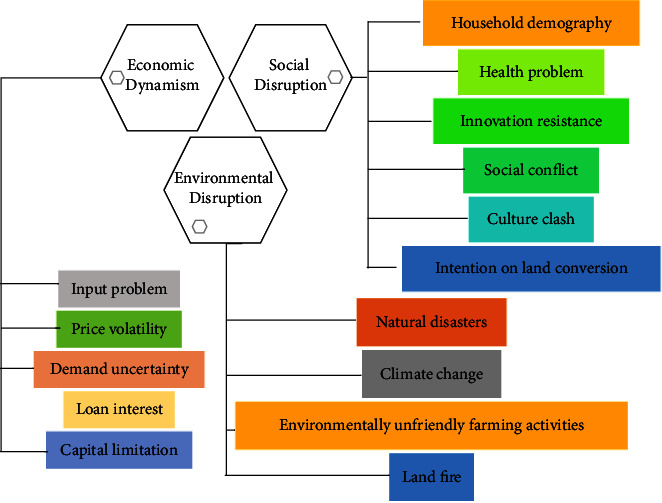
Disruption mapping on the agricultural system (source: constructed by the authors from the literature, 2022) [7–9].

Numerous studies succeed to identify social disruptions in agricultural subjects among others, demographic problems, deadly epidemics, resistance, social domination, and cooperation or attachment (Figure 1) [7, 10, 11]. Research in China's rural areas proved that agricultural depopulation, young people migration, labor migration to the nonagricultural sector, farm income decreasing, and associated social (political status) and psychological changes (gentrification and the feminization of agriculture) have reduced the resilience level of rural people [12, 13]. Furthermore, resistance could be defined as what Curry et al. concluded in their study as the view of modern farming can corrupt indigenous values and practices [14]. This perspective can be a threat to agricultural technology or innovation adoption. Social domination can be explained as the social structure form that robust cultural infrastructure with its own rules and values that regulate the moral behavior of its members. This structure can cause social conflict and cultural clashes with modern values [7]. Cooperation, including contract farming, in many ways, provides more advantages than its disadvantages. However, in several cases, this form of relationship could be extremely bound and harm smallholders if there is an unfair relationship between farmers and companies or institutions. Vamuloh et al. (2020) revealed that small farmers abstained from contract farming primarily due to unfavorable contract requirements [15]. He furtherly explained that contract farming is an exploitative practice that lacks equity.

The next social disruption variable that is predicted to have a significant impact on smallholder plantation resilience is the intention of farmers to convert their farming land [16]. Conversion of farmland can disrupt the sustainability of agriculture. In the case of plantation crops, land conversion can reduce the supply of raw materials, increase prices, and lead to scarcity [17]. From the farmer's perspective, this intention is a driving factor for low productivity because farmers tend to ignore their farming [17, 18]. The farmers tend to transform their agricultural livelihood into a nonagricultural which can offer them a higher income and make them face difficulties in maintaining farming on the remaining agricultural land [18].

In recent studies, environmental disruption has been framed in several forms, including natural disasters (earthquakes, floods, droughts, pests attack, and plant diseases), climate change, environmentally unfriendly cultivation habits, and land fires (Figure 1) [19, 20]. These forms of environmental disruption are also experienced by farmers in Indonesia [21]. The environmental disruption caused by natural events cannot be fully controlled by humans, including earthquakes. Bengkulu is one of the Indonesian provinces that are frequently disturbed by earthquakes [22, 23]. The next environmental disruption factor that has a significant influence on the agricultural sector is climate change [8, 24]. Newly, climate topics are the most popular research themes, particularly in agricultural concerns [25–33].

The environmental disruptions that arise due to human negligence are also considered to be very disruptive, such as land burning and environmentally unfriendly farming activities. Nevertheless, it can still be overcomed by providing education to farmers. Land clearing activities by burning vegetation have an impact on land fire incidents and air pollution [21]. Then, cultivation activities could damage the environment because of the excessive use of chemical substances [19].

Although the plantation businesses in Indonesia are mostly conducted by smallholding farmers, they remain to function as market-oriented businesses. Consequently, they cannot be separated from economic dynamism which can affect business performance (Figure 1). Moreover, several economic disordered circumstances could be the cause of the existence of social or environmental disruption. The first problem that is frequently faced by smallholding farmers in developing countries, including Indonesia, is farmers' affordability to input price, fertilizers availability, and small farmers' accessibility to subsidized fertilizers [34, 35]. Secondly, agricultural product price volatility is well-known as a major disruptive circumstance, and this has had an enormous impact on smallholders [9, 36]. Several impacts that are present because of this problem are a reduction in the usage of production input [37], decreasing income [38], and land conversion [39, 40]. Consequently, it drives farmers to perform an adaptation to their farming activities [36, 38]. The next economic dynamism that can harm a smallholder farmer is demand uncertainty. This problem was claimed by Czekaj et al. as a part of market distortion [9]. Czekaj et al. further explained that lack of capital potentially has a direct impact on farmers' resilience [9]. This limitation of financial resources forces farmers to lend money from another loan institution (formal or informal). This loan commonly requires high interest, and it can be another problem that must be faced by farmers.

As a negative incident, disruption has been considered to harm smallholder plantations' resilience. For the less resilient farmer, even slight changes or disruptions can be devastating. As resilience weakens, it needs a progressively smaller external event to cause catastrophe [41]. Consequently, the degradation of the environmental systems quality and changes in social structure increases the possibility for smallholders to become less resilient [9, 42].

The term resilience was firstly introduced by C.S. Holling in the ecological framework [43]. According to his paper, “Resilience and Stability of Ecological Systems,” he revealed the difference between stability and other conditions that indicate the level of ability of the systems to permeate changes. Resilience was identified as the degree of determination of a system and its capacity to captivate changes and perturbations and still preserve the same intercourse between variables or parameters and populations [43]. It means that resilience is a right of the system, and the result is the resilience or extinction of the system itself. Then, resilience develops into the realm of a more comprehensive system, namely, how ecology relates to social systems. Carpenter et al. (2001) defined it as “socio-ecological system resilience” [44]. Resilience was constructed in various concepts, including as a metaphor related to sustainability, dynamic models' property, and measurable quantification of socio- and ecological systems (SES) [44–46]. Carpenter further explained that the concept of resilience has begun to be widely used in numerous kinds of interdisciplinary work related to the interaction between humans and nature, including agricultural systems.

In the agricultural sector, FAO specifies resilience as the ability to prevent disasters and crises as well as to anticipate, absorb, accommodate, or recover to become more timely, sufficient, efficient, and sustainable behavior [41]. This includes protecting, reestablishing, and improving food and agricultural systems under threats that affect agriculture and food safety [41].

A number of experts try to break down resilience into several dimensions of ability or capacity [47]. Resilience is acknowledged as adaptability [24, 48, 49], recoverability (preventive and protective) [50–52], anticipation [50–54], and innovation level [54–57]. While organizational theory approaches resilience measurement by assessing the ability of systems to detect, respond, and adapt to disturbances [58] and defend themselves in the midst of a challenging environment and then recover from the after-effects [53]. In several studies, adaptability capacity is indicated by systems' experience with natural disasters, transformation in farming activities and resource adjustment and transition [59–61]. Meanwhile, recovery capacity is indicated by robustness, growth, and management [41, 47, 62]. Anticipation capacity is represented by preparedness [63, 64], protection [52], and succession [26]. Then, the innovation level is indicated by initiative, creativity, and entrepreneurship [55]. Resilience's operational indicators have acknowledged slight attention in the literature. In measuring a system's resilience, the researcher must determine which disruption is attractive [44].

**Figure 2:**
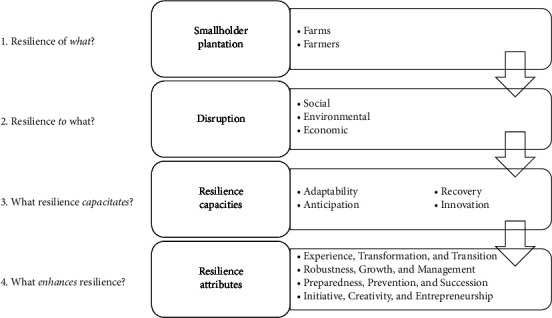
The framework of farming system resilience assessment (authors' modification) [67].

A more in-depth study by Carpenter et al. [44] and Bennett et al. [65], in Cheng et al. (2019), describes resilience in several more detailed and measurable aspects, such as (i) resilience to what (which explains events that can disrupt the system); (ii) resilience of what (which describes the resilience of identity or system); (iii) resilience at what (which shows the scale of environmental conditions around the system); (iv) resilience due to what (which describes the cause of the system to be more resilient); and (v) indicators of resilience (the variables which are considered and capable of measuring the resilience level) [66]. The Carpenter approach model also has been adopted by Meuwissen who formulated a framework for measuring agricultural system resilience [67]. In his work, Meuwissen explained that the framework was constructed to measure resilience to specific challenges as well as a farming system's capacity to handle the unknown, uncertainty, and shock. The framework also provides indicators to assess the performance of system functions, resilience capacities, and resilience-enhancing attributes. Capacities and attributes refer to adaptive cycle processes of agricultural practices, farm demographics, and risk management [67]. Meuwissen followed the three analytical steps of Carpenter (2011) by adding two more aspects, namely resilience capacities and resilience enhancement. This study tries to adopt the resilience approach in four aspects, namely “resilience of what,” “resilience to what,” “what resilience capacitates,” and “what enhances resilience” (Figure 2).

**Figure 3:**
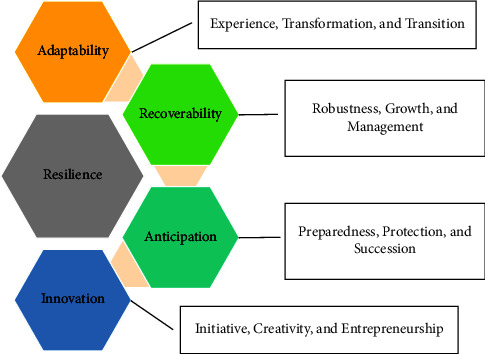
Multidimensional resilience approach (source: constructed by the authors, 2022).

From those views, four main dimensions were commonly used as the benchmark of system resilience, including agriculture, namely, adaptability, recoverability, anticipation, and innovation level. However, these four dimensions mostly were studied partially. This study tries to combine the four dimensions in one frame. As a result, a comprehensive approach can be used to measure the resilience level, its capacities, and the indicators of resilience capacities of the smallholder plantations (Figure 3).

Indonesia is known as one of the biggest plantation producers in the world. Indonesian plantation industries contribute the most national income for the country after mineral resources (oil, petroleum, and coal) [68]. Three priority commodities are highly produced, such as oil palm, coffee, and rubber. However, most of the plantation businesses are owned and generated by smallholders. Smallholder plantation business was defined as the cultivation of plantation crops outside the form of a corporate, such as those cultivated by individuals without a business license or under a household management system [68]. This form of business is synonymous with ownership of limited resources, such as low education, narrow land tenure, limited capital, low bargaining power, conventional cultivation behavior, limited access to technology and market information, and unprofessional business management [69, 70]. Furthermore, the ethical trading initiative (ETI) outlines that most smallholder farmers are very dependent on labor in the family [71]. Like other agricultural businesses, plantations are also inseparable from particular characteristics of agricultural products, which are seasonal, bulky, and time-consuming (gestation period between planting and harvesting time) [72]. Those characteristics make smallholder plantation businesses, particularly in Indonesia, more vulnerable to social and environmental disruptions and tend to be less resilient. Therefore, this study tries to answer how social and environmental disruption affect the resilience level of smallholder plantations in Indonesia.

## 2. Materials and Methods

### 2.1. Literature Review

This study is an empirical study. However, in determining the four dimensions and both resilience and disruption indicators we used a brief literature review approach. Since this study uses comprehensive approaches to learning about social and environmental disruptions and smallholder plantations' resilience, we accomplished the study by reviewing papers by following this step: (i) identifying and mapping social and environmental disruption related to the agricultural system, including smallholder plantations; (ii) searching general (including agriculture) papers with resilience as the keyword (smallholders resilience, organizational, agriculture, farmers, system, and others); (iii) identifying what capacity they used to explain the resilience level; (iv) mapping the capacity and its indicators; and (v) deciding the best capacities and the indicators that are relevant to this research.

### 2.2. Research Location and Data Collection

**Figure 4:**
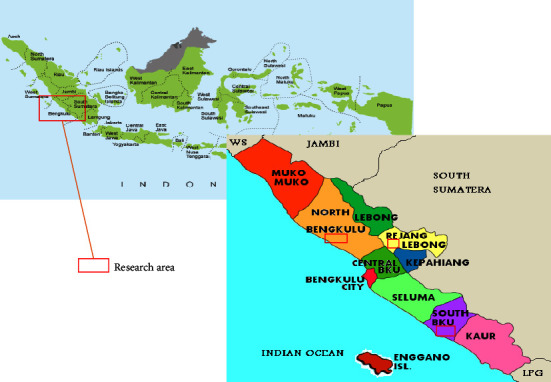
Map of Bengkulu, Indonesia, showing research location (authors' compilation, 2022) [73, 74].

Bengkulu Province was selected as the research area because, based on BPS data, this province is one of the poorest provinces in Indonesia. About 60% of the population are depending on their livelihood in the agricultural sector, and most of them are smallholders. There are about 350 thousand plantation farmers in this region [68]. This research was conducted in six villages from three districts in Bengkulu Province, Indonesia, such as Rejang Lebong, South Bengkulu, and North Bengkulu district (Figure 4). These locations were selected because of their commodity characteristic differences. South Bengkulu is well known as an oil palm producer. North Bengkulu has the largest area of rubber trees and production in the province. Rejang Lebong is located in the highland district of Bengkulu Province which is suitable for coffee plants.

The data used for this research were collected by face-to-face survey. The survey obtained 360 completed and structured questionnaires. 360 selected plantation farmers must follow these criteria: (i) the plantation must be their main farming activities and household income generating, and (ii) the plantation must consist of productive crops. Main farming means that the plantation cultivation area is wider than other commodities, and the time spent at plantation is longer than other activities. Those criteria were questioned in advance before the survey was conducted.

### 2.3. Resilience and Disruption Measurement

12 indicators have been studied and chosen to represent the smallholder plantations' resilience dimensions, such as adaptability, recoverability, anticipation, and innovation. These items were delivered from the literature review. 16 indicators were formulated in the questionnaire to indicate social disruption, and 12 indicators were expressed to indicate environmental disruptions. Statements stated for the resilience and disruption were on a 5-points Likert scale (5 = strongly agree; 4 = agree; 3 = neutral tend to agree; 2 = disagree; and 1 = strongly disagree) [75]. The smallholder plantations' resilience was analyzed by a multidimensional approach, which can be calculated by the following equation:

(1) $$\begin{array}{c} {RPS}_{n}=\overset{i}{\underset{i=1}{\sum }}{ACap}_{n}\text{,} \end{array}$$

where RPSn is the score of smallholder plantation' resilience of respondent *n*, ACap_*n*_ is the total average score of each dimension capacity of respondent *n*, and *i* is the dimension capacity, which is the adaptability, recoverability, anticipation, and innovation [76]. The dimensions, variables, and indicators are explained in Table 1.

### 2.4. Model Estimation

The main question of the study is how do social and environmental disruptions affect the resilience level of smallholder plantations? The smallholder plantation's resilience is quantified in binary and analyzed by the probit model. This analysis adopted Levine's resilience measurement approach [51]. He explained that resilience can be assessed by a probability model. The various measurement appearances cannot be spoken of as a constituent of resilience but only its likelihood predictors [51]. Then, the smallholder plantations' resilience is classified as being more or less resilient. Since there is no global standard to classify resilience level, this study tries to approach a resilience measurement that the resilience level of one respondent is relative to the other responders within the sample population. In other words, as the RPS binary score is based on whether a smallholder's response falls above/below the mean of the sample, the smallholder in the surveyed population is among the more or less resilient only compared to the others involved in the survey. The score 1 is given if the RPS score of the smallholder plantation is more than the mean and will be identified as a more resilient smallholder. Then, if the RPS of the smallholder plantation score is less than or the same as the mean, the smallholder will be scored 0 and grouped as less resilient. The estimation formula to measure the impact of social and environmental disruptions on smallholder plantations' resilience was adopted from resilience measurement by Levine [51] and the framework of disruption and resilience by Sanchis & Poler [77]. The estimation model can be formulated as follows:

(2) $$\begin{array}{c} {RPS}_{n}={HOD}_{n}\beta_{1}+{HEP}_{n}\beta_{2}+{IRE}_{n}\beta_{3}+{SOC}_{n}\beta_{n}+{CUC}_{n}\beta_{5}+{IOC}_{n}\beta_{6}+{NAD}_{n}\beta_{7}+{CLC}_{n}\beta_{8}+{EUC}_{n}\beta_{9}+{LDF}_{n}\beta_{10}+{INP}_{n}\beta_{11}+{PVO}_{n}\beta_{12}+{DUC}_{n}\beta_{13}+{LOI}_{n}\beta_{14}+{CAP}_{n}\beta_{15}+{ɛ}_{n}\text{,} \end{array}$$

where RPS_*n*_ is the smallholder plantation resilience of respondent *n*, *β* is the explaining variables coefficient, and *ε* is the error of the model. According to the literature review, the disruption events are expected to have a significant impact on lowering smallholder plantations' resilience.

## 3. Results and Discussion

### 3.1. The Resilience of Smallholder Plantations

Smallholder plantations' resilience is conceptualized as the capacity for adaptation, recovery, anticipation, and innovation. Each dimension was represented by several relevant indicators. The smallholder plantations in Bengkulu Province have a good recoverability capacity (Table 2). The most recoverable smallholders are coffee farmers, who are classified as having a very good level of recoverability. The recoverability capacity is indicated by robustness, growth, and management. Smallholders' robustness describes how farmers manage business pressure; growth defines their willingness to recover; and management indicates how farmers manage their resources. Farmers explained that they were confident about the continuity of their plantation business. The existence of family support strengthens them during hardship moments, and their faith in God helps them to recover from failures/losses. In contrast, in Zimbabwe, the researcher reported that smallholding and poor farmers hardly recovered from failure, despite the fact that they have received aid programs from the government [62].

Plantation farmers in Bengkulu Province are classified as innovative smallholders. The most innovative farmers are coffee smallholders (very innovative). Innovation capacity was indicated by initiative capacity, creativity level, and entrepreneurship. The research reveals that farmers have the ability to decide business affairs independently and quickly and to conduct actions on initiative, not by others' orders. They are also grouped as “creative farmers,” which is indicated by the ability to find a new way of overcoming farm problems and to design new ideas to run the business. Moreover, the capacity of entrepreneurship was indicated by goal arrangement, confidence level, leadership, motivation in expanding the business, and risk management. Futemma explained that smallholding farmers have the ability to cope with some structural constraints through innovation and entrepreneurship [55].

Overall, the further finding shows that the coffee plantation smallholders in Bengkulu Province are classified as adaptive farmers. Meanwhile, the oil palm and rubber farmers are grouped as less adaptive small farm holders. The farmers argued that they have less experience with catastrophes and diversification in farming activities. Most oil palm farmers explained that they only cultivated oil palm trees on their land. They did not implement a multicrops strategy. About 63% of rubber farmers and 47% of oil palm farmers explained that they cultivate rice on different land areas. However, they revealed that rice farming is only for subsistent food needs, not for commercial purposes. On the contrary, the coffee farmers described having an alternative crop to cover the cost and loss of coffee farms. This finding is in accordance with Wang et al. (2022). They revealed in their study that farmers adopting multiple cropping strategies are more adaptive to climate change problems (heat stress) [78]. The capacity of adaptability was indicated by catastrophe experiences, transformation (farm business diversification), and transition (resources adjustment). Experience toward catastrophe is explained by the intensity of natural disaster experiences, the existence of prevention efforts toward natural disaster incidents, and the ability of farmers to adapt to every natural disaster incident. Diversification of farming activities is described by conducting a multicrops strategy and utilizing another side-crop yield for the plantation. Whereas resource adaptability is explained by preparing production inputs (seeds, fertilizers, and other inputs) independently, maximizing the utilization of family labor, using self-sufficient organic materials, optimizing existing technology, and minimizing dependency on external resources.

Table 2 clarifies that the lowest capacity in describing the resilience level of smallholder plantations is anticipation capacity. The plantation farmers in the research area were identified as less anticipatory smallholders. The anticipation capacity was represented by three indicators, such as the existence of precultivation arrangements, agricultural protection schemes, and plantation successors. Precultivation planning was expressed by the arrangement of scheduled and structured planning for farming activities, action in preparations before cultivation, preparing an alternative strategy to face the risk of disaster or disruption, and creating a backup plan to anticipate crisis or disruption. The farming protection effort was explained by the participation of farmers in agricultural insurance, the availability of reserved funds, and the prevention effort. In addition, the succession effort is indicated by encouraging the successor to pursue the family's business, providing the children with an agricultural education background, and involving the children in farming activities. No farmers are joining an agricultural insurance scheme. Fadhil et al. (2021) explained in their study that the implementation of agricultural insurance in Indonesia still challenges various barriers [79]. Smallholding farmers only have reserved funds to anticipate unpredictable farming costs and cover any losses. Only oil palm farmers have involved their children in plantation activities. Most farmers exposed that they have asked their children to study outside of the village, and most of them did not learn about agriculture as their educational background.

**Figure 5:**
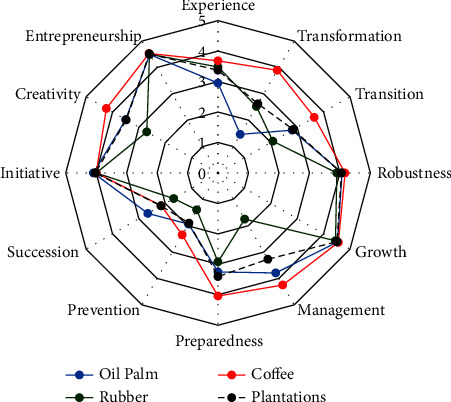
Smallholder plantations' resilience by indicators (source: constructed by the authors based on primary data from field survey, 2021).

Figure 5 illustrates that the most experienced and transformative smallholders are coffee farmers. They are above the average score of smallholder plantations' experience and transformation in Bengkulu Province, whereas oil palm farmers have the lowest score of experience towards catastrophe and transformation capacity. The rubber smallholders are in the lowest level of transition among plantation farmers in the research location. The transition represents the capacity of farmers in resource adjustment. Robustness, growth, and management describe the capacity of recoverability. The three groups of farmers have a good recoverability capacity. It means that the smallholding farmers in Bengkulu Province are able to manage any pressure in farm activities, they have faith to survive, and they also have implemented good resource management (unless rubber smallholders). Moreover, the result indicates a willingness of farmers to recover from the business downturn. Overall, smallholder plantations scored more than 4 of 5 on this indicator. The rubber smallholders were identified as farmers who have the poorest plantation management. Their ability in resource maintenance is at the lowest rate, with a score of less than 2 of 5. This indicator is explained by performing the following actions: replacing damaged plants regularly, conducting plant maintenance intensively, and improving soil conditions after flood, landslides, or other natural disasters.

The coffee farmers execute cultivation preparation more intensively than other farmers. Their prevention effort is also better than oil palm and rubber farmers. However, their succession effort is lower than oil palm farmers but still higher than rubber farmers. According to the field survey during the interview, the rubber farmers revealed that their children decided to study out of the village and learn in nonagricultural schools or colleges. Otherwise, the oil palm farmers exposed that their successors were involved in farm activities and schooled near the village. The three groups of plantation farmers are initiative farmers. The coffee farmers and oil palm farmers were categorized as creative smallholders, whereas rubber smallholding farmers were less creative. Overall, the smallholding plantation farmers in Bengkulu Province have a good entrepreneurship mentality.

Since this research uses a binary model, the score will be transformed into binary, 1 and 0. The smallholder who has a score under or the same as the average RPS score is classified as a less resilient smallholder, and the smallholder who has a score above the average score is classified as a more resilient smallholder.

**Figure 6:**
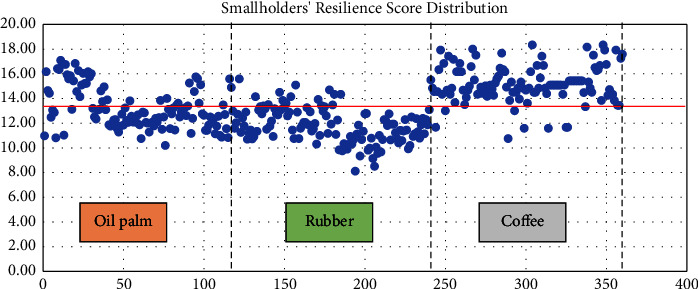
The resilience score of smallholder plantations in Bengkulu province (source: constructed by the authors based on primary data from field survey, 2021). Note. The red line is the average score of RPS (13.38). A number of respondents is 360.

Overall, the result research found that 192 smallholder plantations in Bengkulu Province, or about 53.33% of the respondents are categorized as less resilient smallholders, and 46.67% of them are above the mean lines of resilience level or categorized as more resilient smallholders.  Figure 6 describes the distribution point of the resilience score among smallholders, including oil palm, rubber, and coffee smallholders. According to this figure, more coffee smallholders are spotted above the average score line (92.5%). Only 7.5% of coffee farmers are categorized as less resilient smallholders. On the contrary, more rubber smallholders are under the mean line. It is just 12.5% of rubber farmers positioned above the average line or classified as more resilient smallholders. Meanwhile, 65% of oil palm farmers have resilience scores below 13.38 or are categorized as “less resilient smallholders.” Based on the resilience score, the coffee farmers are classified as the most resilient smallholders among the other smallholder plantations in Bengkulu Province.

### 3.2. Social and Environmental Disruption and Its Impact on Smallholders' Resilience

Based on the literature review, the variables of social disruption that have an impact on smallholder plantations' resilience are household demographic conditions, epidemics and family health, farmers' resistance to change, social problems, cultural clashes, and the intention of farmers on land conversion. The study revealed that almost 60% of farmers agreed with the existence of the demography problem, but only 30% of them explained that this problem affected their farming, such as the activities and income changes. This disruption was indicated by (i) the movement of family members (children/relatives) to the city (it can reduce the workforce in the family to run a plantation business), and (ii) only a few family members can work together in farm activities (lack of productive age of family members). This result is in line with the statistical test in Table 3 that the demography problems have no significant impact on lowering the resilience level of smallholder plantations in Bengkulu Province.

To identify the disruption of the epidemic and family's health problems, this research used two main indicators, such as the COVID-19 pandemic and the health status of the head of family and family members. About 50% of farmers stated that they faced this problem. However, only 30% of them expressed that this disruption influenced their plantation. The statistical result shows that the effect of the epidemic and family's health problems is not significant. Afterward, the variable of farmers' resistance to change is also not significant. Even though 90% of smallholders confessed that they are resistant to change, only 50% of them stated that this perturbation affected their plantation. In addition, the impact of social problems (social conflict and culture clashes) on smallholder plantations' resilience is also statistically not significant. On top of that, the farmers claimed that there is no culture clash in their village.

**Figure 7:**
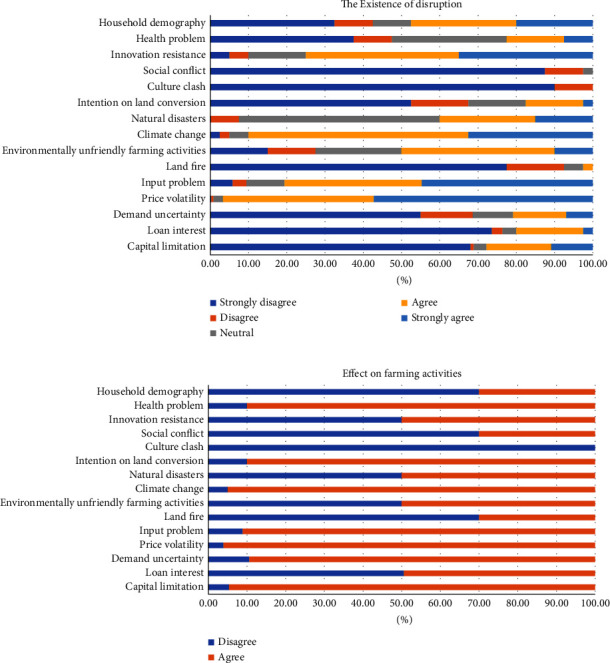
The existence (a) and the effect (b) of social, environmental, and economic disruption (source: constructed by the authors based on primary data from field survey, 2021). The questions or statements (2-scale: agree and disagree) of the effect of each disruption event on farming activities were questioned to farmers who have answered at least a score of 3 (yellow color/neutral) in the statements about the existence of each disruption. The statements/questions of the effect are related to changes in farming activities habits or income.

The last social disruption variable that was expected to have a significant impact on lowering smallholder plantations' resilience is the farmer's intention on land conversion.  Figure 7 describes that more than 30% of farmers agreed with the existence of this intention, and 90% of them claimed that it impacted their farming activities. According to Table 3, the results show that the variable of intention on land conversion has a significant impact on lowering smallholders' plantation resilience. This result is in line with the hypothesis. Conversion of agricultural land can disrupt the sustainability of farming. In the case of plantation crops, land conversion can reduce the supply of raw materials, increase prices, and lead to scarcity. From the farmer's perspective, the intention to convert plantation land is a driving factor for low productivity because farmers tend to ignore their farming. It was normally caused by decrease in income, price reduction [17], land value increase, and family or community encouragement [80]. However, in our research, we did not estimate how much time it will affect social disruption. We used a 5-Likert scale (1 is strongly disagree – 5 strongly agree); a full explanation has been added to Section 2. To gain farmers' responses about what they felt and did, this study used these several statements: (1) the plantation business that is carried out does not provide great benefits for family life; (2) there is a desire to replace the commodity currently being cultivated with another commodity; (3) cultivating other plants can provide better welfare; (4) there is a desire to sell plantation land; and (5) the problem of intention to land conversion makes you adjust in managing your plantation business.

Based on the farmer's perspective, further findings show that the most disruptive incident in environmental disruption is climate change. 95% of farmers agreed with the existence of this environmental disruption, and 95% of them feel the impact on their plantation business activities (Figure 7(b)). Farmers explain that climate change incidents, particularly heat stress, contributed to the changes in their farming activities in the last several years. The heat problem was identified as the most stressful disturbance. This problem was also discovered in the Northern Ghana's smallholder rural farmers [81]. The heat stress problem in the agricultural sector is highly vital according to farming activities which are executed outside and under the sun. While farming, farmers could fall victim to heat stress. Hydrant explained that farmers die from heat-related illness at a rate 20 times higher than any other type of worker in the U.S [82]. Furthermore, besides affecting humans, heat stress has also impacted wheat production in India. This study recommended improving irrigation water-use efficiency [25]. Those facts are supported by the statistical result that the climate change variable significantly influences the lowering of the resilience level of smallholder plantations (Table 3). This result is in accordance with other findings which have confirmed that climate change has significantly negatively affected the resilience of agricultural businesses [8, 83, 84]. This finding is logically accepted because the sustainability of the agricultural industry, especially the cultivation sector, is almost completely dependent on natural conditions.

More than 90% of farmers explained that natural disaster exists (Figure 7(a)). Bengkulu is well-known as one of the Indonesian provinces which experiences more earthquakes than other regions in the country. Based on the field study, floods and droughts are infrequent. These natural disasters had an impact on smallholder plantation businesses in Bengkulu Province. However, only 50% of farmers confessed that this disruption affected their farming activities (crop failure, difficulties in the harvesting and marketing processes, and disrupting the distribution of agricultural production facilities (fertilizers, pesticides, etc.)). According to the binomial probit model analysis, the impact of this variable on smallholders' resilience is not significant (Table 3). Generally, in Bengkulu Province, those types of natural disasters mostly impact residential areas.

Furthermore, 85% of farmers admitted that they did not follow the cultivation methods recommended by field officers. Some statements representing this disruption are (i) do not know how to perform environmentally friendly cultivation; (ii) the application of fertilizers, pesticides, and plant diseases controllers is not under the recommendations of the local extension worker; and (iii) the application of fertilizers, pesticide, and plant diseases controllers is executed excessively. Table 3 confirms that disordered farming implementation statistically has a significant negative effect on smallholder resilience. The existence of this variable increases the possibility of smallholder plantations in Bengkulu Province becoming less resilient. Excessive use of chemical substances could cause the degradation of soil quality and result in decreased productivity [19].

The next variable that has no significant impact on smallholder plantations' resilience is land fires. Farmers explained that land fires in their area rarely occur. There are only a few farmers (less than 10%) who have experience in this incident. In Indonesia, land fires are the biggest disaster in the plantation sector, especially in oil palm. However, these incidents mainly occur in peatlands. The distribution of this soil type is in the provinces of Riau, South Sumatra, and most of the plantation areas on the island of Kalimantan [21].

The farms' resilience has been frequently explored in the field of socio-ecological term [27, 28, 45, 59, 85, 86]. However, it progressively embraces economic dimension which associated with social and ecological systems [9, 46, 56, 87–89]. The result shows that the most significant negative impact of economic dynamism on smallholder plantation resilience is capital limitation. 31% of the respondents confessed that they are desperate to face this problem, and 95% of them claimed that this condition affects their plantation business. Czekaj et al. explained that lack of financial support can be harmful for small farmers to survive [9]. The further finding proves that price volatility of the plantation commodities and demand uncertainty also have a significant negative effect to farm resilience. Price volatility was clearly noticed by many researchers as a major problem for agricultural product [36–39]. Hu and Rahman stated in their study that decreasing in output price has an immense disturbance on smallholders [35]. In this study, the demand uncertainty is indicated by the rejection of farmers' products by processing plant due to lack of quality or factory over capacity. The result shows that 31% of farmers experienced this problem, and 90% of them agreed that this circumstance affects their plantation.

Moreover, the input problems (scarcity and expensive input) have a significant negative effect to the resilience level. The result figures that more than 95% of smallholder farmers faced scarcity of subsidized fertilizers and expensive price of other inputs, 100% of less resilient smallholders suffered because of these difficulties. FAO explained in their report that smallholder farmers in less developed and developing countries are struggling with input problems [69]. Meanwhile, loan interest has no significant impact on smallholder resilience in Bengkulu Province. This result is supported by the fact that more than 75% of farmers claimed that they are never involved in any capital loan, and only 49% of farmers agreed that this problem affects their small farm business.

## 4. Conclusions and Policy Implications

The research mainly purposes to explore the impact of social and environmental disruptions on the resilience of smallholder plantations in Bengkulu Province, Indonesia. The result concludes that more than half of the respondents are less resilient. In this study, social disruptions are conceptualized as negative consequences of farmers' behavior on their livelihood on the plantation. Environmental disruptions are identified as incidents, whether from natural causes or human activities, which have a negative influence on the plantation and empirically affect the plantations' resilience. The social disruption which has the most enormous impact on farming activities and income is a health problem. It is followed by intention on land conversion, innovation resistance, demography problem, and social conflict. Nevertheless, there is no culture clash in the research area. Meanwhile, in environmental disruption, climate change was identified by farmers as the most considerable disruptive event on their plantations. Then, it is followed by environmentally unfriendly farming activities, natural disasters, and land fires. The intention of land conversion, climate change, and environmentally unfriendly farming habits statistically increased the possibility of smallholder plantations in Bengkulu becoming less resilient. Moreover, the economics dynamisms which have a significant impact on lowering smallholder plantation resilience in Bengkulu Province are scarcity of input, price volatility, demand uncertainty, and capital formation.

Because climate change is one of the most considerable disruptions and significantly negatively affects smallholder plantations' resilience, it is essential to draw the right and relevant policy implications from this main problem. Implementing mitigation strategies [90, 91], organizing climate change information centers [86, 92], and the adaptability reinforcement of smallholder plantations due to climate distress [93] are expected to minimize the impact and enhance farmers' business resilience. The other significant environmental disruption is environmentally unfriendly farming activities. To overcome this problem, the government must concentrate on implementing intensive training and extension programs to educate farmers about how to perform the best agricultural practices [85]. The possible training programs are climate-smart agriculture, agricultural organic use, or sustainable agricultural performance (e.g., RSPO or ISPO for Indonesia oil palm plantations). Whereas, in the social disruption, the intention on land conversion attracts more attentions. The government must regulate the price and disordered market problems. The strategies that could be implemented are providing selling price incentives [94, 95], establishing advanced processing plants [96], and organizing export-import regulations and policies [97–99] for plantation commodities, particularly oil palm, rubber, and coffee. The expected return of the processing crops is a market certainty and better price, so the farmers tend to eliminate their intentions of land conversion. Regardless of whether there will be another social disruption, further research needs to be carried out.

Furthermore, to overcome the input problems (scarcity of subsidized fertilizers and overpriced nonsubsidized fertilizers), some recommendations that could be the solution are providing fertilizers storage in the nearest region of the farmers and are implementing better distribution management systems. Since the price volatility was resulted by market conditions, the possible policy is monetary incentive for farmers or establishment of advanced processing factory in local area to stabilize the demand and commodities market price. Some farmers experience a rejection of product from processing company due to lack of quality, so the best way out of this problem is delivering education to farmers about the importance of quality assurance during cultivation and harvesting time. While to eliminate the capital limitation problem, some farmers have performed a multiple-crops strategy and created local funding among farmers' group members to avoid bank loans. These strategies need to be adopted by other farmers who have limited access to formal loan institutions.

## Figures and Tables

**Table 1:** Variables used description and measurement of the empirical model.

Variables	Acronym	Indicators and measurement of the variables
Smallholder plantation resilience	RPS	Binary (1 = more resilient; 0 = less resilient) If RPS ≤ mean, it is categorized as less resilient; and RPS > mean, it is categorized as more resilient
Adaptability capacity	AB	Experience (encounter natural disasters/catastrophes or other natural incidents) Transformation (farming activities diversification strategy) Transition (resources adjustment ability)
Recoverability	RV	Robustness (pressure management capacity) Growth (willingness to recover) Management (resources maintenance)
Anticipation level	AN	Preparedness (risk mitigation strategy and precultivation planning) Prevention (agricultural protection scheme and reserved fund) Succession (regeneration planning)
Innovation level	IN	Initiative (the ability to initiate and implement an action independently) Creativity (the use of original ideas in the plantation activities) Entrepreneurship (the ability on managing the plantation and taking on business risks)
If the average score of each capacity ≤ absolute median, it is categorized as less; and if the average score of each capacity > absolute median, it is categorized as: adaptive/good recoverability/anticipatory/innovative smallholder. The absolute median is 3 The variables “adaptability,” “recoverability,” “anticipation,” and “innovation” are the four dimensions of resilience		
Social disruption		The negative consequences of people'/farmers' behavior on their livelihood for the plantation
Household demography	HOD	Family member migration Limitations of the productive family members Labor conversion to the nonagricultural sector
Health problem	HEP	The COVID-19 pandemic Health problems of household head and family member
Innovation resistance	IRE	The confidence in existing farming Conventional farming systems from generation to generation
Social conflict	SOC	Horizontal conflict intersociety Land conflict intersociety The conflict between people and the company Land proprietor Class of social status
Culture clash	CUC	The local norm which disallows innovation adoption
Intention on land conversion	IOC	The low economic value of plantation production Conversion intention to other commodities Land vending
Environmental disruption		The incidents, whether from natural or human activities causes which have a negative influence on the plantation
Natural disasters	NAD	Disaster incidents frequency Infrastructure and crops damaged The difficulties in harvesting and selling Pest outbreaks and crops diseases
Climate change	CLC	Climate change experience Heat stress Flood or drought incidents due to climate change
Environmentally unfriendly farming activities	EUF	Knowledge limitation about good agricultural practices Exaggerating in use of fertilizers
Land fire	LDF	Land fire frequency Land fire causes Land firing habit during land preparation
Economic dynamism variables:		
Input problem	INP	Indicates the difficulties of farmers in reaching recommended number of agricultural inputs, including fertilizers (particularly subsidized fertilizer)
Price volatility	PVO	Explained by the experience of plantation farmers about the price situation during the last 1-year period
Demand uncertainty	DUC	Indicated by the rejection of plantation products (fresh fruit bunch, rubber, or coffee) by wholesalers/middlemen or manufacturing companies. The rejection could be because of low quality (under company requirements) or out of capacity of the factory
Loan interest	LOI	Explained by the level of loan interest rate according to farmers' experience (categorized as high and low-rate interest)
Capital limitation	CAP	Indicated by the level of availability of internal sources of capital owned by farmers
The social and environmental disruptions, and the economic dynamism were categorized as: not very disruptive (score 1.00–1.80); less disruptive (score >1.80–2.60); quite disruptive (>2.60–3.40); disruptive (>3.40–4.20); and very disruptive (>4.20–5.00)		

Source. Constructed by authors from the literatures [41, 51, 67, 75].

**Table 2:** Resilience level of smallholder plantations in Bengkulu province, Indonesia.

Dimensions/indicators	Ave. score	Std. dev.	Category	Ave. score	Std. dev.	Category	Ave. score	Std. dev.	Category	Ave. score	Std. dev.	Category
	Oil palm			Rubber			Coffee			Plantation		
Adaptability	2.40	0.6370	Less adaptive 2.41	2.69	0.7709	Less adaptive 2.69	3.74	0.5454	Adaptive 3.74	2.95	0.8714	Less adaptive 2.95
Experience	2.95	0.8397		3.48	0.7187		3.68	0.7550		3.37	0.8303	
Transformation	1.46	0.7436		2.52	1.6902		3.89	1.1796		2.62	1.6083	
Transition	2.81	0.9967		2.08	0.6684		3.65	0.7700		2.85	1.0411	

Recoverability	4.13	0.5179	Good 4.13	3.37	0.3926	Good 3.37	4.33	0.4714	Good 4.33	3.94	0.6215	Good 3.94
Robustness	4.07	0.6253		3.90	0.4392		4.17	0.6595		4.05	0.5919	
Growth	4.53	0.4487		4.45	0.4649		4.56	0.5666		4.51	0.4968	
Management	3.79	0.8366		1.75	0.8816		4.25	0.5538		3.27	1.3320	

Anticipation	2.62	0.4368	Less anticipatory 2.62	2.00	0.6071	Less anticipatory 2.00	2.85	0.5127	Less anticipatory 2.85	2.49	0.6342	Less anticipatory 2.49
Preparedness	3.25	0.7580		2.92	0.8208		4.04	0.6801		3.40	0.8888	
Prevention	1.94	0.7292		1.40	0.6909		2.35	0.6060		1.90	0.7805	
Succession	2.66	0.7724		1.68	0.9341		2.15	1.0387		2.16	1.0031	

Innovation	4.03	0.6389	Innovative 4.03	3.73	0.6478	Innovative 3.73	4.25	0.5468	Innovative 4.25	4.01	0.6471	Innovative 4.01
Initiative	4.09	0.9526		4.00	0.9958		4.00	0.9958		4.03	0.9798	
Creativity	3.50	1.0073		2.70	1.4327		4.23	0.4976		3.48	1.2206	
Entrepreneurship	4.49	0.4311		4.50	0.4663		4.53	0.5218		4.51	0.4735	

Resilience score average	13.38				Less resilient smallholders	53.33%						

Respondents	360				More resilient smallholders	46.67%						

Source. primary data from field survey (2021).

**Table 3:** Probit model estimation results in the impact of social and environmental disruptions on smallholders' resilience.

Variable	Coefficient	Std. error	*z*-statistic	Prob.	Marginal effect
HOD	0.0195	0.0475	0.4118	0.6805	1.0197
HEP	0.0741	0.0832	0.8911	0.3729	1.0770
IRE	0.2736	0.0954	2.8671	0.0041	1.3149
SOC	0.4649	0.3056	1.5213	0.1282	1.5924
CUC	0.0333	0.3305	0.1009	0.9196	1.0339
IOC	−0.3848	0.0849	−4.5330	0.0000	0.6804^*∗∗∗*^
NAD	0.0935	0.0855	1.0944	0.2738	1.0981
CLC	−0.2307	0.1093	−2.1097	0.0349	0.7939^*∗∗*^
EUF	−0.4877	0.1055	−4.6210	0.0000	0.6139^*∗∗∗*^
LDF	−0.0974	0.1369	−0.7115	0.4768	0.9071
INP	−0.1329	0.0761	−1.7472	0.0806	0.8755^*∗*^
PVO	−0.2852	0.1227	−2.3238	0.0201	0.7517^*∗∗*^
DUC	−0.1308	0.0636	−2.0568	0.0397	0.8774^*∗∗*^
LOI	0.1904	0.1240	1.5355	0.1247	1.2099
CAP	−0.1638	0.0580	−2.8228	0.0048	0.8488^*∗∗∗*^
Constanta	2.8077	0.7818	3.5913	0.0003	
Log-likelihood	−197.7762				
LR *x*^2^	101.9124				
Prob (LR statistic)	0.0000				
Respondents	360				

^*∗∗∗*^, ^*∗∗*^ and ^*∗*^ approve that it is significant at 1%, 5%, and 10%, respectively.

## Data Availability

The data are available upon reasonable request to the corresponding author.
